# The actual conditions of traditional Japanese Kampo education in all the pharmacy schools in Japan: a questionnaire survey after the enforcement of the new national 2015 core curriculum

**DOI:** 10.1186/s12906-018-2368-5

**Published:** 2018-11-08

**Authors:** Yoshinobu Nakada, Makoto Arai

**Affiliations:** 1 Department of Kampo Medicine, Tokai University School of Medicine, Isehara, Kanagawa Japan; 2 Medical and Pharmaceutical Society for WAKAN-YAKU, Kanagawa, Japan

**Keywords:** Core curriculum, Education, Kampo medicine, Pharmacy school, Questionnaire survey

## Abstract

**Background:**

To investigate the present status of Kampo education, which has still not been elucidated, after the introduction of the new core national curriculum of 2015 into nationwide pharmacy education, in all 74 pharmacy schools in Japan.

**Methods:**

A postal questionnaire survey was conducted from August 2015 to January 2016. The completed questionnaires were returned by mail. Web-based syllabi were also investigated to ascertain the detailed lecture curricula in each school. Descriptive analyses were conducted without statistics.

**Results:**

A total of 74 questionnaires were collected (response rate, 100%). In 2015, the numbers of clinical Kampo classes as required subjects during the 6 years of regular pharmacy school education ranged from 0 to 36 (median, 13; mean, 11.8 ± 7.6). Of the 74 schools, 49 schools (66%) provided Kampo education from a clinical standpoint. Pharmacists employed in pharmacies and physicians taught most of these classes. The major problems to be solved first are: selecting and retaining teachers to teach clinical Kampo medicine (43 of 74 schools, 58%), preparing standard textbooks (37 schools, 50%), and improving the environment for practical Kampo training (30 schools, 41%).

**Conclusions:**

Curricula for teaching Kampo medicine significantly differ at each of the 74 Japanese pharmacy schools. In addition to selecting teachers who can adequately teach clinical Kampo medicine, improving training environments, and nationwide standardization of the curricula and textbooks are critical.

**Electronic supplementary material:**

The online version of this article (10.1186/s12906-018-2368-5) contains supplementary material, which is available to authorized users.

## Background

Japanese traditional medicine (Kampo medicine) was uniquely developed, especially in the Edo period (1603–1868), after having been introduced from China centuries prior to that [1, 2]. It was the primary medicine until the end of the Edo period. Western medicine, however, gradually took over during the Meiji period (1868–1912) and has continued as the primary form of medicine in Japan until the present day. The large factor of this change was that the national licensing examination for physicians only asked questions related to Western medicine. Under this long slump in Kampo medicine, Kampo theories (especially those in herbal medicine) were only handed down by a very few pharmacists and physicians in their own offices. Finally, the situation in clinical sites gradually began to change due to the listing of Kampo medicines in the national health insurance program in 1967. Over the subsequent years, Kampo therapy has become increasingly popular among Japanese people [3–5]. Moreover, 70 to 90% of Japanese physicians regularly prescribe Kampo medicine in their medical practices according to clinical evidence and reports of mechanisms of action and/or by following guidelines from modern Western medicine [6–8]. The education of Kampo medicine, however, was still largely inadequate until the early days of the twenty-first century.

The curricula in Japanese pharmacy schools changed significantly in 2006, when they extended their 4-year program to 6 years and increased their clinical medicine classes. Since then, Kampo medicine education has been definitely incorporated into the curricula of all the pharmacy schools. Furthermore, in 2015 the newly revised core curriculum has been added, in which concepts, education of uses, side effects, adverse events, and interactions with Western medicines of Kampo medicine have been emphasized. In addition, topics on the way to catch patients’ patterns, systematic classifications of Kampo formulae, Kampo medicines in contemporary medicine, management of Kampo drugs in pharmacies, and education for patients have all been newly added, which are namely educational points from the clinical side, compared with the prior curriculum which emphasized education of crude drugs, active ingredients of crude drugs, and drug efficacy evaluations. Traditionally, however, pharmacists have been able to sell Kampo medicines in their drugstores without prescriptions. One hundred forty-eight kinds of Kampo formulae are currently approved by the Japanese national health insurance system. If patients are treated within these Kampo medicines and want to use the national health insurance, they have to ask their physicians and get a prescription. On the other hand, if patients opt not use the insurance, they can go directly to pharmacies or hospitals providing treatment which is not covered by the national health insurance. Namely, pharmacists can sell Kampo medicine directly, over-the-counter (OTC), and Kampo formulae of crude drugs to their customers in their pharmacies, even though the Japanese national health insurance does not cover it. Moreover, pharmacists have the right to ask physicians (indeed, they have the professional responsibility to ask), if they notice any mistakes, or points of concern, in any prescriptions they handle. Therefore, it is imperative that pharmacists have standard and adequate knowledge of Kampo medicine. E.g., especially the mineralocorticoid action of Glycyrrhiza containing glycyrrhizin and its interactions with diuretics, and the sympathomimetic effect of the Ephedra herb containing ephedrine are some of the crude drugs which physicians and pharmacists should remember. Similarly, they have to know their contraindications and they have to identify the excess overlapping of crude drugs among multiple Kampo prescriptions from multiple hospitals [9]).

Interestingly, it was not until 2001, for the first time in Japanese medical schools, that the national guidelines on medical education core curriculum approved the education of Japanese traditional medicine. Since then, however, among all the 80 Japanese university medical schools, an increasing number of them have integrated Kampo medicine into their curricula [1]. According to our previous study [10], which shows the outcomes of a questionnaire survey of all the Japanese medical schools in 2011, 98% of them conducted at least one Kampo medicine class, and 81% taught Kampo medicine on the basis of the traditional Japanese theory. The most outstanding problems to be solved first discovered from the survey were selecting and retaining teachers to teach Kampo medicine (65%), curricula standardization (63%), and preparation of standardized textbooks (51%). In the pharmacy education in Japan, however, to our knowledge, no such survey has ever been done after introducing the new national core curriculum of 2015. The aims of this study are to elucidate the present conditions of Kampo education in all the Japanese pharmacy schools and, from these data, to put together basic materials to teach Kampo medicine in the near future.

## Methods

We conducted a postal questionnaire survey of the present status of Kampo education in all 74 pharmacy schools (17 national and public schools and 57 private schools) in Japan from August 2015 to January 2016. The questionnaires (see the Additional file 1) were distributed by postal mail to the pharmacy directors in every university pharmacy school first and subsequently by them to the teachers who teach Kampo medicine.

There were two main categories of questions. One category was regarding, “The present status of Kampo related education in your pharmacy school,” which asked about the number of units offered, the types of classes (lectures and/or workshops), the contents of the education, and its concepts. The other category was, “Opinions regarding Kampo related education,” which asked the teachers detailed questions about their opinions of Kampo medicine. The questions were about the present opinions about the number of lectures, the education about crude drugs and their extracts, the basics of Kampo medicine, the clinical uses of Kampo medicine, and the side effects, adverse events, and interactions with Western medicines. Questions were also asked about the necessity of standardized textbooks, the necessary education about the future of Kampo medicine, and any other related problems that have to be solved as soon as possible. In addition to the questionnaire, we referred to Web-based syllabi to investigate the detailed lecture curricula in each school. In the question about the items that should be added to Kampo education, a “circle” answer (indicating an item that should be required in future curricula) was calculated as 1 point, and a “triangle” answer (indicating an item that should be an elective) was calculated as 0.5 points.

To prepare the questionnaires for the present study, we mainly used the semantic differential method, which is often applied in psychological research, to assure their validity [11]. Completed questionnaires were returned by postal mail. To increase the response rate, directors of any schools that did not return responses were asked again for their completed questionnaires by postal mail. We conducted descriptive analysis without statistics to show everyone that this survey accurately reflects the present status of Kampo education in the pharmacy schools throughout Japan. All the responders gave written informed consent to participate in this study.

This survey was approved by the Institutional Review Board for Clinical Research of Tokai University School of Medicine and conformed to the principles of the Helsinki Declaration. The survey was also approved by the Medical and Pharmaceutical Society for WAKAN-YAKU, the official Japanese scientific organization dealing with Japanese traditional medicine.

## Results

### Study population

A total of 74 questionnaires were collected, meaning that responses were obtained from every pharmacy school in Japan (response rate, 100%).

### Percentages of graduates becoming clinical pharmacists after graduating from Japanese pharmacy schools

Seventy-three pharmacy schools (99%) answered this question. After graduating from Japanese pharmacy schools, the percentages of graduates becoming clinical pharmacists were as follows. Twenty-five schools (34%) answered more than 90%. In 16 schools (22%) it was from 70 to 90%, 11 schools (15%) was from 50 to 70%, 10 schools (14%) was from 30 to 50%, 6 schools (8%) was from 10 to 30%, and 5 schools (7%) was less than 10% (median, 70–90%) (Table 1).

**Table 1:** Percentages of graduates becoming clinical pharmacists after graduating from Japanese pharmacy schools

Proportions	Schools
≥90%	25 (34%)
70 to 90%	16 (22%)
50 to 70%	11 (15%)
30 to 50%	10 (14%)
10 to 30%	6 (8%)
< 10%	5 (7%)
Total	73

### Present status of Kampo related education in Japanese pharmacy schools

The length of 1 class (lecture or workshop) ranged from 60 to 105 min, with a median of 90 min, for which 71 schools answered (96%). Most schools (60 schools, 85%) offered 90 min per unit. The class lengths per unit offered at other schools were: 60 min (1 school), 70 min (5 schools), 75 min (1 school), 80 min (3 schools), and 105 min (1 school).

**Fig. 1:**
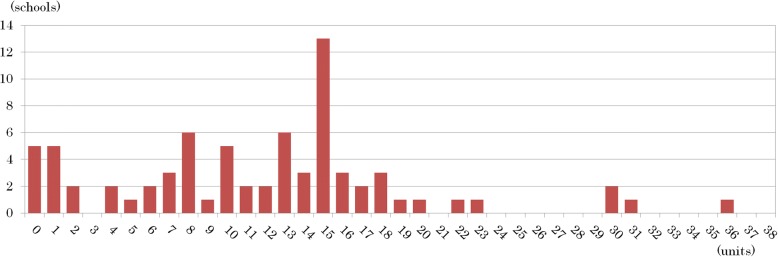
The number of clinical Kampo classes, except for pharmacognosy, pharmaceutical botany, natural products chemistry, complementary and alternative medicine other than Kampo medicine and/or practical training, as required subjects during the 6 years of pharmacy schools as of 2015 (range, 0–36; mean, 11.8 ± 7.6; median, 13 classes). These data were extracted in detail from Web-based syllabi and by referring to the questionnaires collected from 74 pharmacy schools

The number of clinical Kampo classes, except for pharmacognosy, pharmaceutical botany, natural products chemistry, complementary and alternative medicine other than Kampo medicine (e.g., acupuncture, acupressure, and qigong), and practical training, as required subjects during the 6 years of pharmacy schools ranged from 0 to 36 classes with a mean of 11.8 ± 7.6 classes and a median of 13 classes in 2015. These data were extracted in detail from Web-based syllabi and by referring to the questionnaires collected from 74 schools (Fig. 1). In addition, 51 schools (69%) offered clusters of consecutive Kampo classes (lectures and/or workshops). On the contrary, however, 5 schools (7%) had no clinical Kampo classes as required subjects, which means that pharmacy students in those schools had no opportunities to receive any clinical Kampo education. One of those 5 schools was in a transitional period of developing its curriculum, so that there were no required clinical Kampo medicine classes in 2015.

**Fig. 2:**
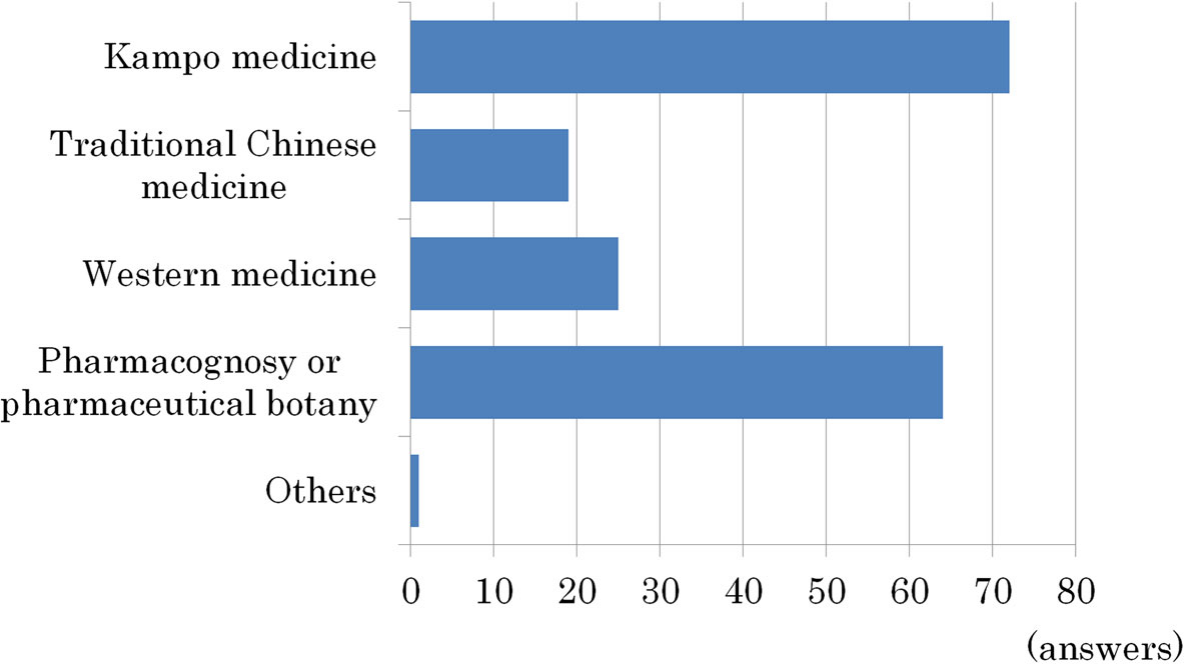
The concept of Kampo related education. A total of 72 schools (97%) were based on Japanese traditional medicine; on the other hand, 19 schools (26%) were based on TCM (traditional Chinese medicine), 25 schools (34%) on Western EBM (evidence-based medicine), 64 schools (86%) on pharmacognosy and/or pharmaceutical botany, and only 1 school was based on the general history of medicine (multiple responses were permitted)

Regarding the concepts in Kampo related education, 72 schools (97%) were teaching Japanese traditional medicine. While 19 schools (26%) were teaching traditional Chinese medicine (TCM), 25 schools (34%) were teaching Western evidence-based medicine (EBM), 64 schools (86%) were teaching pharmacognosy and/or pharmaceutical botany, and only 1 school was teaching the general history of medicine (multiple responses) (Fig. 2).

**Fig. 3:**
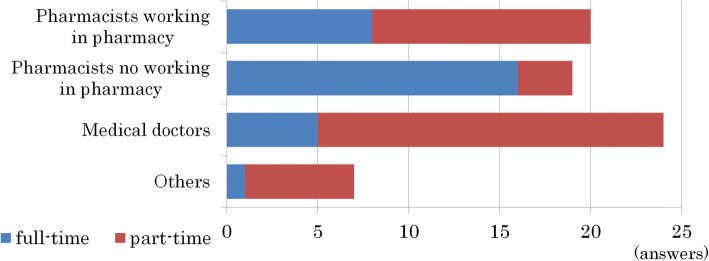
Teachers’ qualifications, of those who taught clinical-based medicine in the pharmacy schools answered by 49 schools (66%). These classes were mostly taught by pharmacists employed in pharmacies and physicians. Thirty-one schools (63%) recruited part-time teachers from among pharmacists employed in pharmacies and physicians. Physicians were recruited in 24 schools (49%)

Forty-nine schools (66%, of all 74 schools) provided Kampo education from a clinical standpoint. These classes were taught most by pharmacists employed in pharmacies and physicians. Thirty-one schools (63%) selected part-time teachers from among those pharmacists employed in pharmacies and physicians and these teachers taught all the classes (lectures and/or workshops) on Kampo related education in 7 of these schools. Physicians taught Kampo medicine in 24 schools (49%). In addition to these specialists, lectures were given by TCM specialists (in 3 schools, 6%), a medical representative (in 1 school), a teacher other than a pharmacist or a physician (in 2 schools), and educational video lectures (in 2 schools) (multiple responses) (Fig. 3). Finally, some Kampo related classes were offered in “hands-on” practical training (in 10 of 66 schools, 15%).

### Opinions regarding the present Kampo related education

**Fig. 4:**
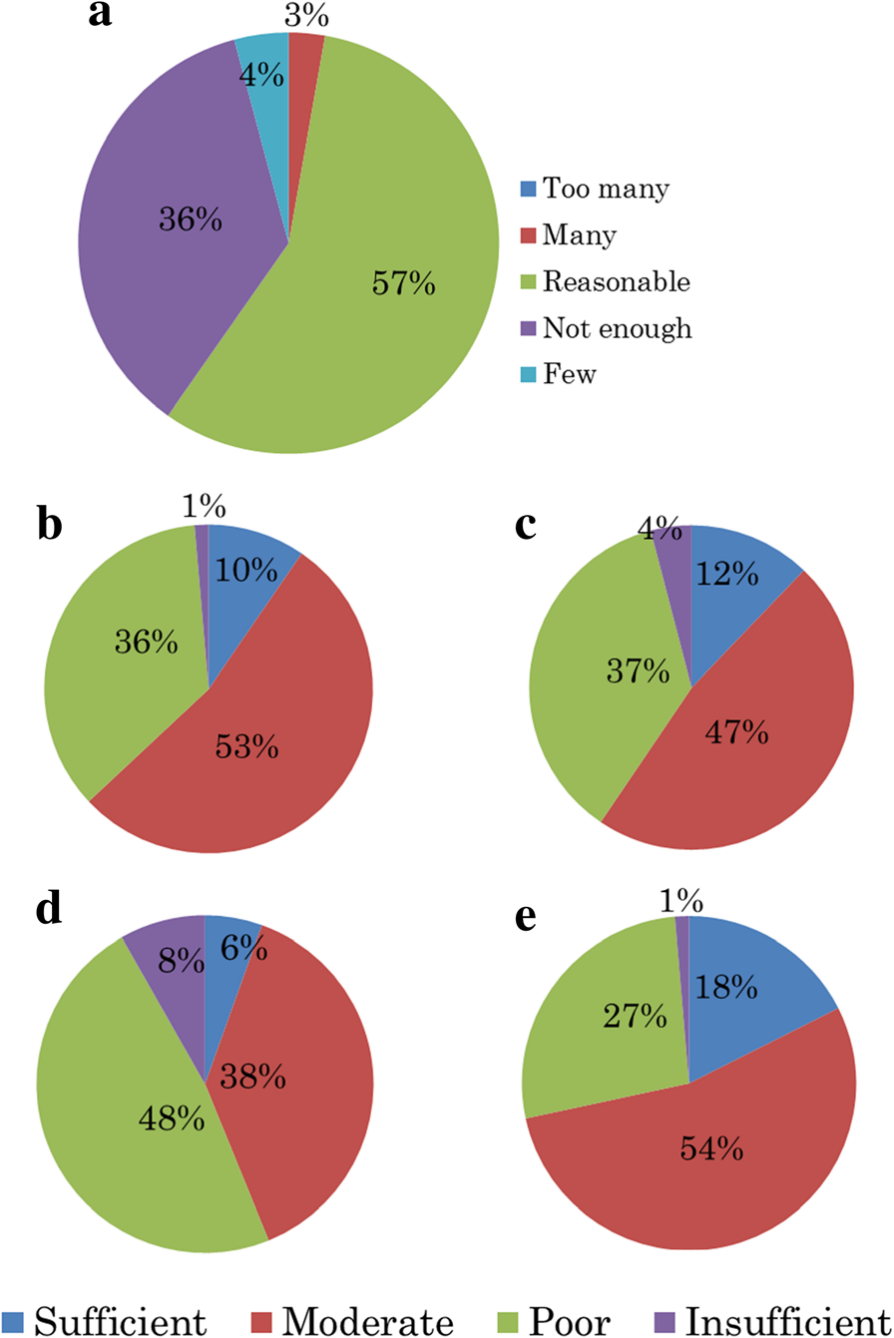
Research results in the satisfaction with present Kampo related education. **a**. The number of lectures in the curricula. Forty-one of 72 schools (57%) answered that the number of lectures in the curricula was reasonable. **b**. Education regarding Kampo related crude drugs and their extracts. Forty-six of 73 schools (63%) answered that the education was adequate. **c**. Education regarding the basics of Kampo medicine (features, technical terms, and core concepts, among others). Forty-four of 74 schools (59%) answered that they were adequate. **d**. Education regarding clinical uses (diagnostic methods, how to “catch” [i.e., diagnose] the patterns of “Sho,” standard prescription process, and the uses of Kampo medicine in contemporary medicine, among others) Thirty-two of 73 schools (44%) answered that the education was adequate but not in 41 schools (56%). **e**. Education of points to be emphasized in Kampo medicine (side effects, adverse events, and drug interactions, among others). Fifty-three of 74 schools (72%) answered that they were adequate

Forty-one of 72 schools (57%) answered that the number of lectures in the curricula was reasonable; however, 29 schools (40%) noted a shortage of lectures (Fig. 4a).

Education about Kampo-related crude drugs and their extracts (Fig. 4b) and about the basics of Kampo medicine (features, technical terms, and core concepts, among others) (Fig. 4c) were reported as being adequate in 46 and 44 schools of 73 and 74 schools (63 and 59%), respectively. The education of clinical applications (diagnostic methods, how to “catch” [i.e., correctly diagnose] the patterns of the “Sho” [the patient’s symptoms at a given moment], standardized prescription processes, and the roles of Kampo medicine in contemporary medicine, among others) (Fig. 4d) were reported as being adequate in 32 of 73 schools (44%) but not in 41 schools (56%). Finally, regarding the education of the points to be emphasized in Kampo medicine (side effects, adverse events, and drug interactions, among others), 53 of 74 schools (72%) answered that they were adequate (Fig. 4e).

### Opinions on the future of Kampo related education

About pre-training of Kampo medicine (i.e., at the undergraduate level), 16 of 74 schools (22%) answered that it should be a required subject, and 53 (71%) thought that it ought to remain an elective subject. However, 5 schools (7%) indicated that it was needless.

Fifty-two of 73 schools (71%) answered that nationwide standardized textbooks were necessary, and 21 schools (29%) answered that standardized textbooks were “especially” necessary. No schools indicated that they were, “Unnecessary.”

**Fig. 5:**
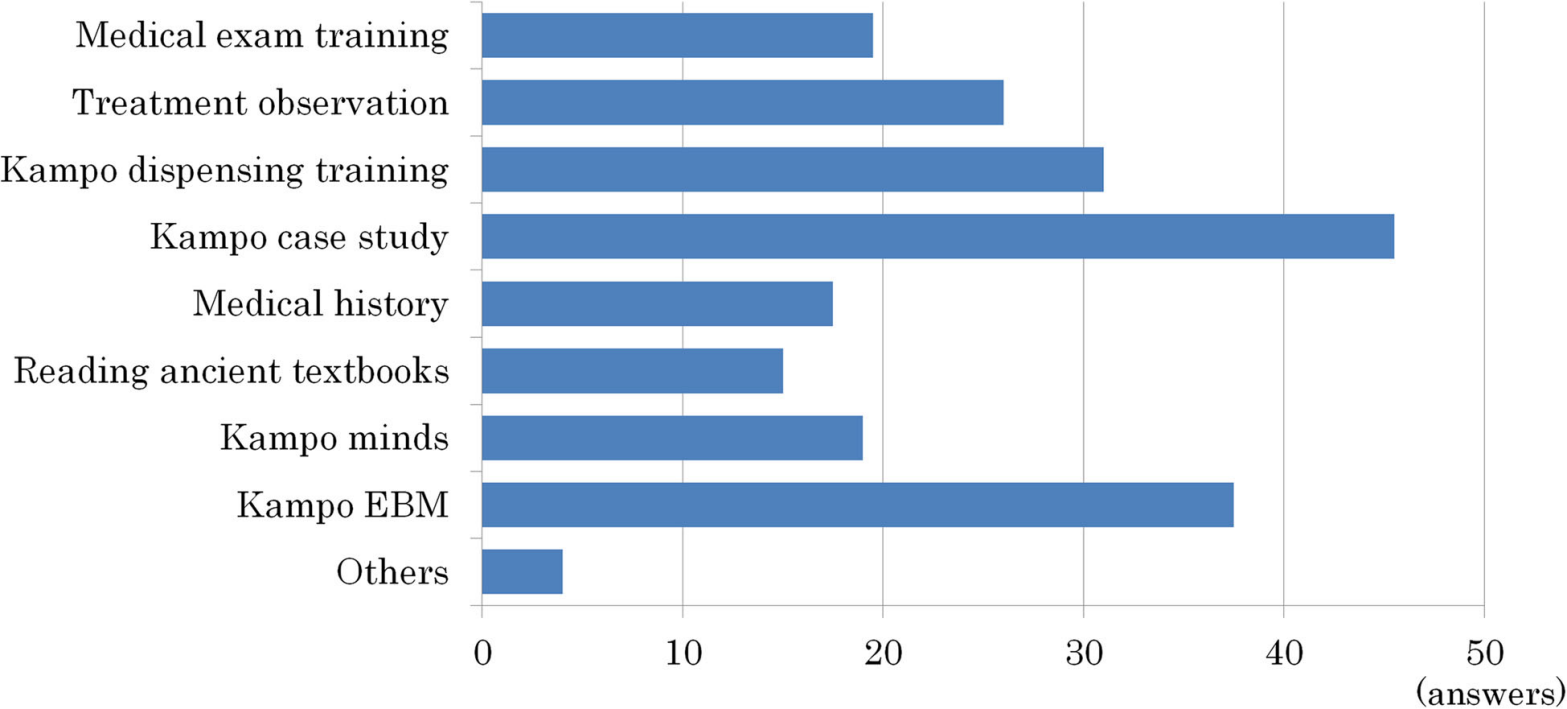
Items that should be added to Kampo related education except for the items that are already listed in the new 2015 national core curriculum (multiple responses, from 74 schools). The most common item noted was case studies (61%), followed by EBM in Kampo medicine (51%), Kampo medicine dispensing training (42%), and real-time observation of medical treatment (35%)

The answers of the items that should be added to Kampo education, except for the items that have already been listed in the new 2015 core curriculum, are given below (multiple responses, from 74 schools). The most common answer was Case studies (61%), followed by EBM in Kampo medicine (51%), Kampo medicine dispensing training (42%), and Observation of medical treatment (35%). These data suggest that many pharmacy schools are interested in improving their clinical Kampo education. “Others” (5%) consisted of crude drug resources, composition of crude drugs and their extracts in Kampo formulae, traditional medicines around the world, and research methods (1 answer each) (Fig. 5).

### The problems with Kampo related education

**Fig. 6:**
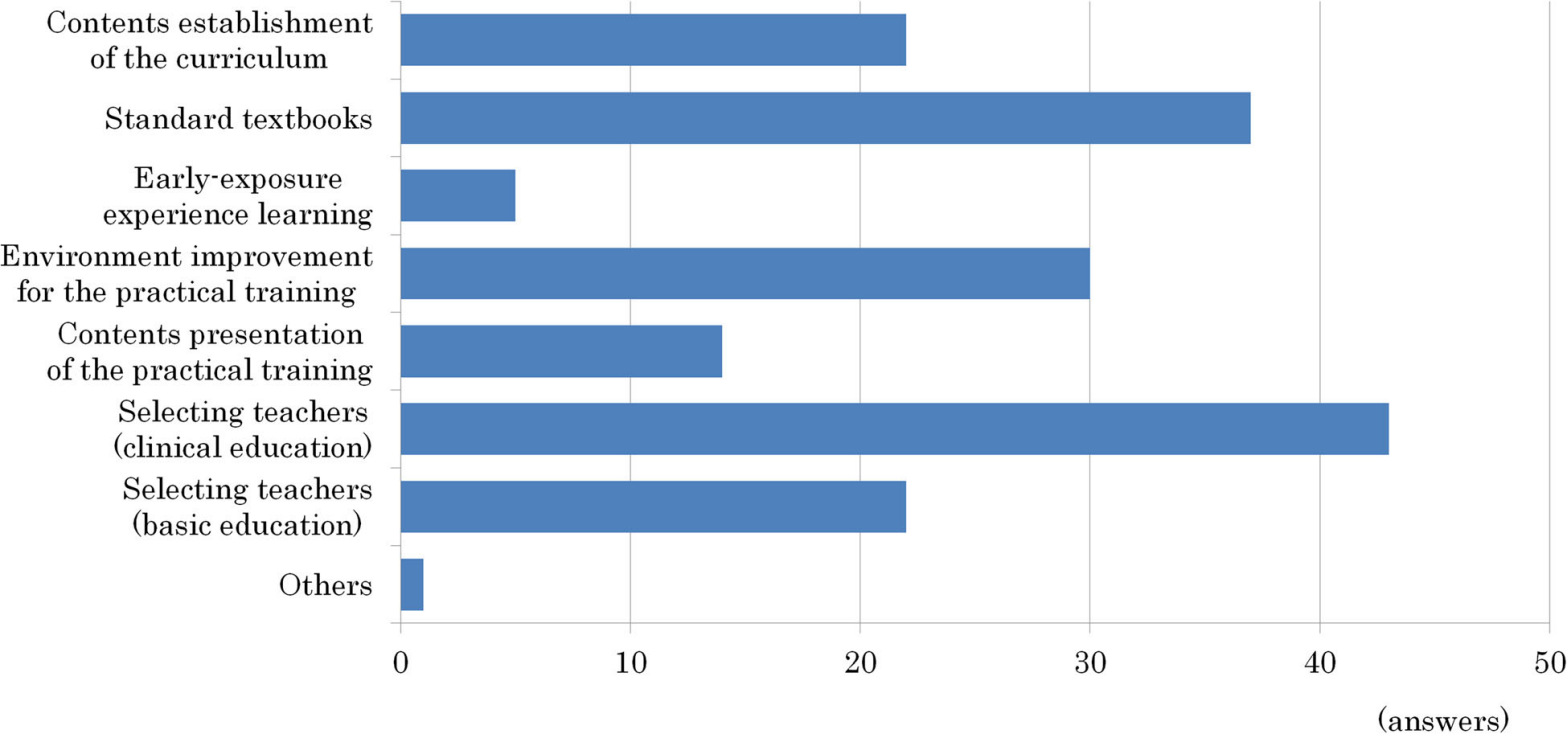
The problems that have to be solved in the Kampo education from 74 schools. Selecting and retaining teachers who can teach Kampo medicine for clinical settings was the most critical problem (43 schools, 58%), followed by preparation of standard textbooks (37 schools, 50%), and improvement of the environment for practical training (30 schools, 41%)

Selecting and retaining adequate teachers to teach clinical Kampo medicine was the most critical problem (43 of 74 schools, 58%), followed by preparation of standard textbooks (37 schools, 50%) and improvement of the environment for practical training (30 schools, 41%). Among others were, “There was a change in teachers’ individual approval of Kampo medicine” (Fig. 6).

## Discussion

We reported on the present status of Kampo medicine education in all 74 pharmacy schools in Japan and the related problems that should be solved as soon as possible as revealed by data acquired from a postal questionnaire survey. We found significant differences in the Kampo medicine curricula among the schools, including the number and length of classes (be they lectures and/or workshops), and the actual contents of the education, among other important issues. Clinical-based Kampo education was largely provided by part-time teachers, who were usually pharmacists employed in pharmacies or physicians. More than half of the schools answered that the education for the clinical use of Kampo medicine was insufficient. As for future outlooks, 93% of the schools answered that pre-training (i.e., at the undergraduate level) in some form of Kampo medicine was necessary, and more than half wanted to incorporate case studies and EBM into Kampo related medical education as required subjects. Finally, “Selecting and retaining teachers to teach clinical Kampo medicine” and “Preparation of standard textbooks” were selected by more than half of the schools as critical problems.

As we previously pointed out, Kampo therapy has become increasingly popular among Japanese people [3–5]. And moreover, 70 to 90% of Japanese physicians regularly prescribe Kampo medicine in their medical practice. [6–8] Concurrently, more extensive scientific evidence of Kampo formulae has been accumulated [12, 13], and their quality and safety have been maintained at higher levels with the progress of Kampo extract formulae [14] resulting in their more substantial integration into Western medicine [15, 16]. In most cases of patients being prescribed Kampo drugs, patients benefited from Kampo medicine when it was used in combination with Western medicine (when they simultaneously received biomedical drugs) [17].

However, there are some points that should be noted when pharmacists sell Kampo medicines. Firstly, when patients buy OTC Kampo medicines, pharmacists must consult with them to help avoid any side effects, adverse events, and/or interactions with any other medications they may be taking. Secondly, pharmacists have the right and the professional responsibility to ask physicians about any uncertainties on the prescriptions. Therefore, pharmacists working in pharmacies have to have general knowledge about the use and any major drawbacks of Kampo medicine.

However, the present survey showed that standardization of Kampo education in pharmacy schools was a long way off and that a few schools provided no clinical Kampo education at all. Presently, many pharmacists, who want to learn more about Kampo medicine, find Kampo education programs themselves, often after getting their national pharmacist license. Similarly, as reported by Arai, et al. [10], Kampo education in medical schools throughout Japan has not been standardized; although, that procedure has begun. To ensure that pharmacists and physicians have the same knowledge about Kampo prescriptions, to help patients and avoid patient confusion, standardization is mandatory. Furthermore, examples of clinical use, including case studies and EBM, should adequately be taught, and preparation of standard textbooks should certainly be a common objective among all the schools. Moreover, standardization of Kampo education and standard textbooks among all the pharmacy schools is essential for the Japanese licensing exam for pharmacists. On the other hand, 7% of respondents answered that pre-training of Kampo medicine was needless. It is considered that the yearly schedule in each school is very busy. However, Kampo pre-training will help new pharmacists work proficiently with Kampo medicine.

Selecting and retaining teachers is one of the big issues in Kampo medicine education. This was revealed to be the largest problem to be solved as soon as possible in Japanese medical education [1, 10]. The present survey showed that more than half of the pharmacists working in pharmacies and physicians were hired as part-time teachers to teach clinical Kampo medicine. However, the other schools employ pharmacists who do not work in pharmacies or other specialists to teach their clinical Kampo medicine classes. This problem requires an immediate solution because even new pharmacists, who have just gotten their licenses, sell Kampo medicines in their pharmacies on a daily basis. Minimal essential clinical knowledge of Kampo medicine should be obtained before their employment in a pharmacy. Therefore we suppose that Kampo education from a clinical standpoint, which is provided by pharmacists employed in pharmacies and physicians who know the current situation in clinical medicine, is required. Cooperation among schools, e.g., teachers sharing and faculty development [10], could be a solution to the problem of the lack of teachers in Kampo education from a clinical standpoint. Thereby, many pharmacy schools will be able to begin teaching Kampo education from the clinical side.

In Kampo education in some medical schools, to attract students, certain techniques have been used, e.g., actual interviews with patients [18], students receiving acupuncture [19], and students undergoing medical examinations given by classmates (diagnosing each other using the “qi, blood, and fluid” system) [20, 21]. On the other hand, experience with crude drugs and crude drug extracts is offered in pharmacy schools [22]. If pharmacy students learn to use Kampo medicine during their first year of training in hospitals, they will become increasingly more interested in Kampo medicine [23]. According to a questionnaire survey targeting students in Kyushu University of Health and Welfare [24], it was revealed that pharmacy students realized the importance of Kampo medicine in contemporary medical settings. At the same time, they indicated that the study of Kampo medicine increasingly becomes more difficult as they advanced in their school years. Students in their fourth through sixth years of pharmacy school wanted to study clinical Kampo medicine through case studies and learn about drug interactions between Kampo and Western medicines. They indicated that they wanted to learn how to provide patients with actual, practical Kampo treatments [24, 25].

Finally, according to a fact-finding survey of Japanese undergraduate education in Sino-Japanese traditional medicine for pharmacy students published in 2002, 10 of 46 pharmacy schools (21%) provided no Kampo education at all, and only 3 schools offered Kampo education as a required subject [26]. In comparison, according to the present survey, although there are more than a few problems remaining to be solved (e.g., selecting adequate teachers and preparing standard textbooks), Kampo medicine education in Japanese pharmacy schools is improving exponentially on a nationwide basis. Many graduates from pharmacy schools choose to work in pharmacies, therefore, Kampo clinical knowledge in contemporary medicine is essential not only for patients’ safety but also for a positive mindset and motivation of pharmacy students and new pharmacists to keep studying and learning about Kampo medicine.

This survey was carried out just after the commencement of the new core curriculum. Therefore, the Kampo educational program at each school may be under revision. Further survey is warranted to elucidate the mature status of Kampo education in pharmacy schools teaching the 2015 core curriculum.

## Conclusion

The educational curricula of Kampo medicine significantly differ nationwide among Japanese pharmacy schools. To solve this problem, in addition to selecting and retaining teachers who can adequately teach clinical Kampo medicine, the preparation of standard textbooks and curricula standardization is especially warranted, and among the many other related problems that must be solved immediately, to ensure that the best Kampo education possible is provided in pharmacy schools throughout Japan.

## Additional file

Additional file 1: Questionnaire on Kampo related education in the 6-year curricula in Japanese pharmacy schools. (DOCX 37 kb)

## References

[1] Motoo Y, Seki T, Tsutani K (2011). Traditional Japanese medicine, Kampo: its history and current status. Chin J Integr Med.

[2] Yu F, Takahashi T, Moriya J, Kawaura K, Yamakawa J, Kusaka K (2006). Traditional Chinese medicine and kampo: a review from the distant past for the future. J Int Med Res.

[3] Yamashita H, Tsukayama H, Sugishita C (2002). Popularity of complementary and alternative medicine in Japan: a telephone survey. Complement Ther Med.

[4] Hori S, Mihaylov I, Vasconcelos JC, McCoubrie M (2008). Patterns of complementary and alternative medicine use amongst outpatients in Tokyo, Japan. BMC Complement Altern Med.

[5] Togo Toshihiro, Urata Shigeru, Sawazaki Kenta, Sakuraba Hinata, Ishida Torao, Yokoyama Kazuhito (2011). Demand for CAM Practice at Hospitals in Japan: A Population Survey in Mie Prefecture. Evidence-Based Complementary and Alternative Medicine.

[6] Oka T (2006). The role of Kampo (Japanese traditional herbal) medicine in psychosomatic medicine practice in Japan. Int Congr Ser.

[7] Terasawa K (2004). Evidence-based reconstruction of Kampo medicine: part-III– how should Kampo be evaluated?. Evid Based Complement Alternat Med.

[8] Muramatsu S, Aihara M, Shimizu I, Arai M, Kajii E (2012). Current status of Kampo medicine in community health care. Gen Med.

[9] Textbook of Traditional Japanese Medicine. Part 1: Kampo. Health and Labour Sciences Research Grant. http://kampotextbook.sakura.ne.jp/pdf/Part1_Kampo_Textbook_of_Traditional_Japanese_Medicine_en.pdf. Accessed 5 Nov 2018.

[10] Arai M, Katai S, Muramatsu S, Namiki T, Hanawa T, Izumi S (2012). Current status of Kampo medicine curricula in all Japanese medical schools. BMC Complement Altern Med.

[11] Osgood CE (1962). Studies on the generality of affective meaning systems. Am Psychol.

[12] Evidence reports of Kampo treatment. 2013. 402 RCT. http://www.jsom.or.jp/medical/ebm/ere/pdf/EKATE2013.pdf. Accessed 5 Nov 2018.

[13] Motoo Y, Arai I, Hyodo I, Tsutani K (2009). Current status of Kampo (Japanese herbal) medicines in Japanese clinical practice guidelines. Complement Ther Med..

[14] Nishimura K, Plotnikoff GA, Watanabe K (2009). Kampo medicine as an integrative medicine in Japan. JMAJ.

[15] Fuyuno I (2011). Japan: will the sun set on Kampo?. Nature.

[16] Watanabe Kenji, Matsuura Keiko, Gao Pengfei, Hottenbacher Lydia, Tokunaga Hideaki, Nishimura Ko, Imazu Yoshihiro, Reissenweber Heidrun, Witt Claudia M. (2011). Traditional Japanese Kampo Medicine: Clinical Research between Modernity and Traditional Medicine—The State of Research and Methodological Suggestions for the Future. Evidence-Based Complementary and Alternative Medicine.

[17] Katayama Kotoe, Yoshino Tetsuhiro, Munakata Kaori, Yamaguchi Rui, Imoto Seiya, Miyano Satoru, Watanabe Kenji (2013). Prescription of Kampo Drugs in the Japanese Health Care Insurance Program. Evidence-Based Complementary and Alternative Medicine.

[18] Takayama S, Ishii S, Takahashi F, Saito N, Arita R, Kaneko S (2016). Questionnaire-based development of an educational program of traditional Japanese Kampo medicine. Tohoku J Exp Med.

[19] Takashi M, Nakada Y, Arai K, Arai M (2016). Educational importance of acupuncture and moxibustion: a survey at the Tokai University School of Medicine Japan. Tokai J Exp Clin Med..

[20] Arai M, Arai K, Hioki C, Takashi M, Matsumoto K, Honda M (2013). Evaluation of medical students using the “qi, blood, and fluid” system of Kampo medicine. Tokai J Exp Clin Med.

[21] Arai M, Arai K, Hioki C, Takashi M, Honda M (2013). Evaluation of Kampo education with a focus on the selected core concepts. Tokai J Exp Clin Med..

[22] Kobayashi Y (2016). Kampo medicine in the new model Core curriculum of pharmaceutical education. Yakugaku Zasshi.

[23] Matsuda H (2016). Approach to teaching Kampo medicine at Kyoto Pharmaceutical University. Yakugaku Zasshi.

[24] Atsumi T, Uehara N, Kawasaki R, Ohtsuka I, Kakiuchi N (2015). Attitudes of pharmacy students at Kyushu University of health and welfare toward Kampo medicine from a questionnaire survey conducted in 2012. Kampo Med.

[25] Kim S, Matsumoto T, Kiyohara H, Hayasaki T, Muranishi A, Hanawa T (2004). Evaluation of Kampo medical education by pharmaceutical students. J Trad Med.

[26] Shoji N (2002). A fact-finding survey of Japanese undergraduate education in Sino-Japanese traditional medicine for pharmacy students. J Trad Med..

