# Supplementary material for: The actual conditions of traditional Japanese Kampo education in all the pharmacy schools in Japan: a questionnaire survey after the enforcement of the new national 2015 core curriculum
Source: BMC Complement Altern Med. 2018 Nov 8;18:297. doi: 10.1186/s12906-018-2368-5 (PMC6225583; doi:10.1186/s12906-018-2368-5)
Supplement: Supplementary file 1 — Questionnaire on Kampo related education in the 6-year curricula in Japanese pharmacy schools. (DOCX 37 kb) [file 12906_2018_2368_MOESM1_ESM.docx]

**Questionnaire on Kampo related education in the 6-year curricula in Japanese pharmacy schools**

**University: _______________________________________________________________________**

**Name and Signature: ______________________________________________________________**

**Department or Section and Position: _________________________________________________**

**Email: ______________________________________**

(To receive the outcome of this study via email)

• “Kampo related education” in this questionnaire includes education of “traditional Japanese medicine” (Kampo medicine), traditional Chinese medicine (TCM), pharmacognosy, and pharmaceutical botany, although the Medical and Pharmaceutical Society for WAKAN-YAKU, the Japan Society for Oriental Medicine, and the Japanese Society of Pharmacognosy basically have opinions distinguishing between Kampo medicine and TCM.

• Please answer all the questions you can regarding the 6-year curricula in your pharmacy school.

• Consent with this survey is indicated by your signature above.

**Present status of Kampo related education in your pharmacy school**

1. What percent of graduates become clinical pharmacists from your pharmacy school?

1. <10%

2. 10–30%

3. 30–50%

4. 50–70%

5. 70–90%

6. >90%

1. How long are classes per unit? ______ minutes
2. Write on the accompanying sheet the name of the classes, school year the classes are offered, the number of units of the classes, if they are elective or required classes, lectures and/or workshops, basic (e.g., pharmacognosy or pharmaceutical botany, among others) or clinical (e.g., Kampo medicine or TCM, among others), and whether teachers are full-time or part-time. One full unit is counted when Kampo related classes exceed half of one unit.

(Continued on page 2)

1. Please indicate the main concepts of medical education at your pharmacy school

(Multiple responses allowed)

1. Kampo medicine (Japanese traditional medicine)

2. TCM

3. Western medicine (evidence based medicine among others)

4. Pharmacognosy and/or pharmaceutical botany (ingredients, classification, among others)

5. Others (Please state specifically. ________________________________________________)

1. Is there any education from a clinical standpoint?

1. Yes → Go on to Question 6.

2. No → Go on to Question 7.

1. What qualifications does the teacher have who teaches clinical-based classes?

Please indicate whether the teacher is full-time or part-time. (Multiple responses allowed)

1. Pharmacist working in a pharmacy (full-time or part-time teacher)

2. Pharmacist no working in pharmacy (full-time or part-time teacher)

3. Medical doctor (full-time or part-time teacher)

4. Others (Please state specifically. ________________________) (full-time or part-time teacher)

1. Is there any Kampo related practical training?

1. Yes

2. No

**Opinions regarding Kampo related education**

• Items that are: 1) basic to Kampo medicine, 2) clinical application of Kampo medicine, and 3) the points to be noted in Kampo medicine in (10) Kampo medicine in medical service are written in the Model Core Curriculum for Pharmacy Education.

**Opinions regarding current Kampo related education except for practical training**

1. What is your opinion about the number of lectures and/or classes offered in the curriculum so that you can achieve your objective in Kampo related education?

1. There are too many.

2. There are many.

3. There are enough–a reasonable number of lectures and/or classes.

4. There are not enough.

5. There are few.

(Continued on page 3)

1. To what degree do you think the education of Kampo related crude drugs and their extracts is adequate?

1. Sufficient

2. Moderate

3. Poor

4. Insufficient

1. To what degree do you think the education of the basics of Kampo medicine is adequate (features, technical terms, and core concepts, among others)?

1. Sufficient

2. Moderate

3. Poor

4. Insufficient

1. To what degree do you think the education of the clinical aspects of Kampo medicine is adequate (e.g., diagnostic methods, how to “catch” [i.e., diagnose] the patterns of “*Sho*,” the actual prescription processes, and the roles of Kampo medicine in contemporary medicine)?

1. Sufficient

2. Moderate

3. Poor

4. Insufficient

1. To what degree do you think Kampo medicine related education is adequate (side effects, adverse events, and drug interactions with western medicine, among others)?

1. Sufficient

2. Moderate

3. Poor

4. Insufficient

1. To what extent do you think clinical Kampo medicine “pre-training” (i.e., at the undergraduate level) is necessary?

1. Necessary as a required subject

2. Necessary as an elective subject

3. Unnecessary

(Continued on page 4)

**Your opinion of the future outlook regarding Kampo related education in pharmacy schools**

1. To what extent do you think nationwide standardized textbooks are necessary?

1. Quite necessary

2. Slightly necessary

3. Hardly necessary

4. Unnecessary

1. What should be further added to future Kampo related education? Draw a “circle” to indicate items you think should be “required” and a “triangle” to indicate those you think should be “elective.” No mark indicates, “Not necessary.” (Multiple responses allowed)

1. Medical examination training (abdominal, pulse, and tongue exam, among others)

2. Observation of medical treatment

3. Kampo medicine dispensing training

4. Case studies in Kampo medicine

5. The general history of Kampo medicine

6. Reading ancient textbooks (“*Shang Han Lun*” and “*Jin Gui Yao Lue*,” among others)

7. Kampo minds (Kampo physicians’ attitudes towards patients and medicine, among others)

8. EBM (evidence based medicine) in Kampo medicine

9. Others (Please state specifically.) ________________________________________________

1. What are the problems that should be solved as soon as possible in Kampo related education? (Multiple responses allowed)

1. Establishing the standard contents of the curriculum

2. Preparation of the standard textbooks

3. Introduction of early-exposure experience-based “hands-on” learning

4. Improvement of the environment for practical training (e.g., teachers’ training)

5. Presentation of the contents of the practical training of Kampo medicine to students

6. Selecting teachers to teach clinical Kampo medicine

7. Selecting teachers to teach basic Kampo medicine

8. Others (Please state specifically.) ________________________________________

_____________________________________________________________________

This is end of the questions. Thank you for your participation.

Correspondence: Makoto Arai, M.D., Ph.D.

Director of Education

Medical and Pharmaceutical Society for WAKAN-YAKU

Professor of the Department of Kampo Medicine, Tokai University School of Medicine

Tel: 0463-93-1121 (Ext. 2249) Fax: 0463-95-3892

Email: [arai@tokai-u.jp](mailto:arai@tokai-u.jp)
